# Pharmacological Mechanisms Underlying Gastroprotective Activities of the Fractions Obtained from *Polygonum minus* in Sprague Dawley Rats

**DOI:** 10.3390/ijms13021481

**Published:** 2012-02-01

**Authors:** Suhailah Wasman Qader, Mahmood Ameen Abdulla, Lee Suan Chua, Hasnah Mohd Sirat, Salehhuddin Hamdan

**Affiliations:** 1 Department of Biological Science, Faculty of Biosciences and Bioengineering, Universiti Teknologi Malaysia, UTM Skudai, Johor 81310, Malaysia; E-Mail: wqsuhailah2@live.utm.my; 2 Department of Molecular Medicine, Faculty of Medicine, University of Malaya, Kuala Lumpur 50603, Malaysia; E-Mail: ammeen@um.edu.my; 3 Metabolites Profiling Laboratory, Institute of Bioproduct Development, Universiti Teknologi Malaysia, UTM Skudai, Johor 81310, Malaysia; E-Mail: lschua@ibd.utm.my; 4 Department of Chemistry, Faculty of Science, Universiti Teknologi Malaysia, UTM Skudai, Johor 81310, Malaysia; E-Mail: hasnah@kimia.fs.utm.my

**Keywords:** *Polygonum minus*, HPLC, UPLC-ESI-MS/MS, gastroprotective mechanisms

## Abstract

The leaves of *Polygonum minus* were fractionated using an eluting solvent to evaluate the pharmacological mechanisms underlying the anti-ulcerogenic activity of *P. minus*. Different *P. minus* fractions were obtained and evaluated for their ulcer preventing capabilities using the ethanol induction method. In this study, Sprague Dawley rats weighing 150–200 g were used. Different parameters were estimated to identify the active fraction underlying the mechanism of the gastroprotective action of *P. minus*: the gastric mucus barrier, as well as superoxide dismutase, total hexosamine, and prostaglandin synthesis. Amongst the five fractions from the ethanolic extract of *P. minus*, the ethyl acetate:methanol 1:1 v/v fraction (F2) significantly (*p* < 0.005) exhibited better inhibition of ulcer lesions in a dose-dependent manner. In addition, rats pre-treated with F2 showed a significant elevation in superoxide dismutase (SOD), hexosamine and PGE2 levels in the stomach wall mucosa in a dose-dependent matter. Based on these results, the ethyl acetate:methanol 1:1 v/v fraction was considered to be the best fraction for mucous protection in the ethanol induction model. The mechanisms underlying this protection were attributed to the synthesis of antioxidants and PGE2.

## 1. Introduction

Peptic ulcer is a common digestive disease and is considered to be a major cause of morbidity and mortality [1]. Scientists have postulated that gastric ulcers are caused by an imbalance between defense mechanisms such as blood flow rate, mucous/bicarbonate production, and endogenous prostaglandin enzymes, and aggressive factors such as stress, hydrochloric acid, *Helicobacter pylori*, smoking, anti-inflammatory drugs, and pepsin production [2–4]. Most research has concentrated on natural antioxidants in plant extracts to cure a variety of ailments, including peptic ulcers. *Polygonum minus* is a plant belonging to the family Polygonaceae and is locally called kesum in Malay [5]. This plant has a nice, sweet aroma and is commonly used by the Malaysian population as a flavoring ingredient. In the last decade, several studies have been reported that *P. minus* possesses high free radical scavenging activity and reducing power using different extracting solvents [6,7]. Researchers have concentrated on *P. minus* in order to identify its phytochemical constituents because it is highly aromatic and rich in essential oils [8]. In addition, as it is not toxic *in vitro* [7] or *in vivo* [9], it can be safely used in humans. Traditionally, *P. minus* has been used to treat digestive disorders. Pharmacologically, *P. minus* has been reported to possess antimicrobial [10], antiviral, and anticancer activity [11].

In the current study, the ethanol induction model was used as it has been reported as a rapid and convenient model of gastric ulcer induction in experimental animals. As well, ethanol is considered to be a necrotizing factor that contributes to the formation of lesions in the gastric mucosa [12]. The mechanism underlying this lesion has been demonstrated to involve damage to the vascular endothelium causing edema formation and lifting, or it may be due to the stagnation of gastric blood flow, resulting in the appearance of hemorrhage, necrosis, and tissue injury [13]. Currently, there are no data available regarding the gastroprotective capacity and the mechanism of action of the fractions obtained from *P. minus*. Following the traditional use of *P. minus* to treat digestive disorders, this study was conducted to evaluate for the first time the pharmacological mechanisms underlying the anti-ulcerogenic activities of the fractions obtained from an ethanolic extract of *P. minus* using different analytical grade solvents.

## 2. Results and Discussions

### 2.1. Effect of *Polygonum minus* Fractions on Gastric Lesions in the Ethanol Induction Model

**Figure 1:**
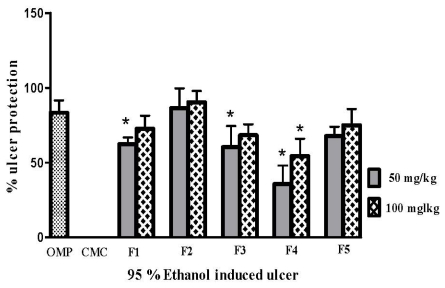
Percentage inhibition of the ulcer area in rats pre-treated with omeprazole 20 mg/kg and *Polygonum minus* fractions (F1, F2, F3, F4, and F5; 50 and 100 mg/kg). Each column expresses the mean ± SEM of *n* = 6 using one-way ANOVA. * *p <* 0.05 *vs.* omeprazole.

**Figure 2:**
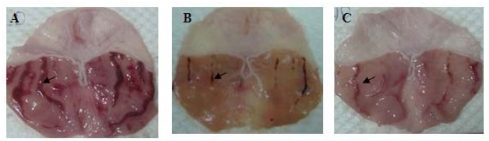

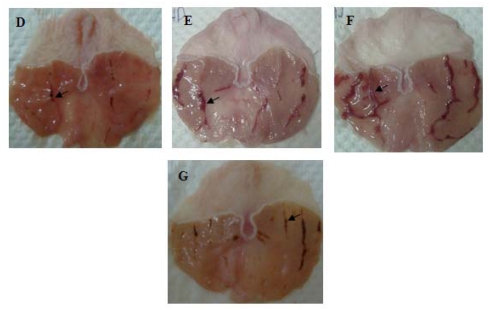
(**A**) Macroscopic evaluation of the gastric mucosa of rats pre-treated with CMC (carboxymethyl cellulose, ulcer control group); (**B**) Omeprazole, 20 mg/kg (positive control group); (**C**) F1 100 mg/kg; (**D**) F2 100 mg/kg; (**E**) F3 100 mg/kg; (**F**) F4 100 mg/kg; (**G**) F5 100 mg/kg (magnification: 1.8×). The black arrows indicate gastric lesions.

**Figure 3:**
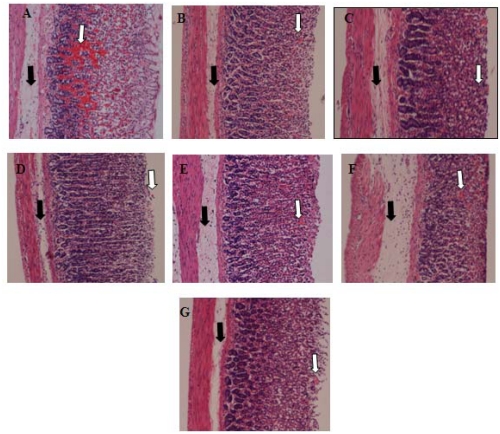
(**A**) Histological evaluation of the gastric mucosa of rats pre-treated with CMC (ulcer control group); (**B**) Omeprazole, 20 mg/kg (positive control group); (**C**) F1 100 mg/kg; (**D**) F2 100 mg/kg; (**E**) F3 100 mg/kg; (**F**) F4 100 mg/kg; (**G**) F5 100 mg/kg. White arrows represent severe disruptions to the surface epithelium and deep mucosa, while black arrows represent leukocyte infiltration and edema in the submucosal layer (H & E stain; 10×).

All the fractions from the ethanolic extract of *P. minus* reduced ulcer area significantly (*p* < 0.05) when compared with the CMC (carboxymethyl cellulose) negative control group presented in Figure 1. It is clear that F4 had a lower inhibition percentage of ulcer areas (54.34% ± 11.56) when compared with the omeprazole positive control group, whereas F2 was the most effective fraction as it inhibition ulcer formation to 90.30% ± 7.49 at the 100 mg/kg pre-treated dose. The gross evaluation shown in Figure 2 showed that rats in the CMC negative control group exhibited extreme injury to the mucosa from ethanol induction (Figure 2A), while pre-treatment with F1, F3, F4, and F5 showed moderate lesions in different ratios (Figure 2C,E–G, respectively). Rats in the OMP pre-treatment positive control group and the F2 group showed a reduction in ulcer area compared to other groups (Figure 2B,D respectively). This result was confirmed after histological examination as shown in Figure 3. In the CMC negative control group, the gastric mucosa was extensively damaged with edema and leukocyte infiltration of the submucosal layer (Figure 3A). The rats pre-treated with F1, F3, F4, and F5 showed markedly better reductions in gastric lesion (Figure 3C,E–G, respectively), while the OMP positive control group and the F2 group demonstrated significantly better mucosal protection with less mucosal damage, edema, and leukocyte infiltration (Figure 3B,D respectively).

The present study focused on fractions of *P. minus*, using different analytical HPLC grade solvents, to evaluate the mechanism of gastroprotection mediated by this plant. Peptic ulcer is postulated to be a multifactor disease, but each factor acts via a particular mechanism to contribute to the aetiology of the mucus lesion [14]. The mechanism of ethanol induction is attributed to the creation of free radicals and an elevation in lipid peroxidation [15], which are considered major agents of inflammation and tissue injuries [16]. In contrast, most plant resources possess markedly high antioxidant and total phenolic compounds that play an important role in the scavenging of free radicals [17,18].

The current medical treatment of peptic ulcer is in general based on the enhancing of mucosal defense against necrotizing effects such as alcohol as well as none steroidal anti-inflammatory drugs (NSAIDs). However, one of the major problems in gastro-duodenal ulcer treatment is that despite the healing rate of 80–100% after 4–8 weeks of therapy with H2 antagonists and proton pump inhibitors, the rate of ulcer recurrence within 1 year after stopping management is between 40% and 80% [19]. Besides, most of these drugs lack effect on other factors involved in ulcer disease and therefore, do not achieve all treatment goals. In addition, it can result in several adverse reactions and expensive. Hence, there is a strong need for more effective and safe anti-ulcer agents [20].

Several beneficial plant extract are used to enhance the mucosal defense mechanism, shift in proton pump, stabilizing surface epithelial cells or interfering with prostaglandin synthesis [21]. Moreover, plant extracts are the important sources for the new drug development due to their greater safety and high antioxidant composition.

In the present study, the results of the gross and histological evaluations showed that ethyl acetate:methanol 1:1 v/v fraction F2 from *P. minus* promoted better inhibition of lesions in the gastric mucosa compared to the other fractions (Figures 1, 2D and 3D). The high content of phenolic compounds, namely gallic acid and coumaric acid, could be the main active compounds of this plant, particularly in F2, which displayed potentially significant ulcer prevention effects upon the stomach and is worthy of further consideration [22,23].

As the incidence of gastric ulcer increases over time, scientists expect it to have a significant effect on the health, patients lives and the economic system of related countries [24] in addition, non steroidal anti-inflammatory drugs NSAIDs have now become a regular prescriptive drug in the family, which is a powerful cause of gastric ulcer. Hence, a widespread search has been necessary to identify new anti-ulcer therapies from natural resources. Several clinical researches have confirmed the gastroprotection activities conferred by plants on patients [25–27]. This study is further providing insights into the future use of herb based drugs as alternative prevention and treatments for gastric ulcer. Accordingly, the phenolic compounds from *P. minus* could be potentially used as a natural drug to protect human mucosa from necrotizing agents.

### 2.2. Effect of *Polygonum minus* Fractions on the Mucus Wall Barrier

**Figure 4:**
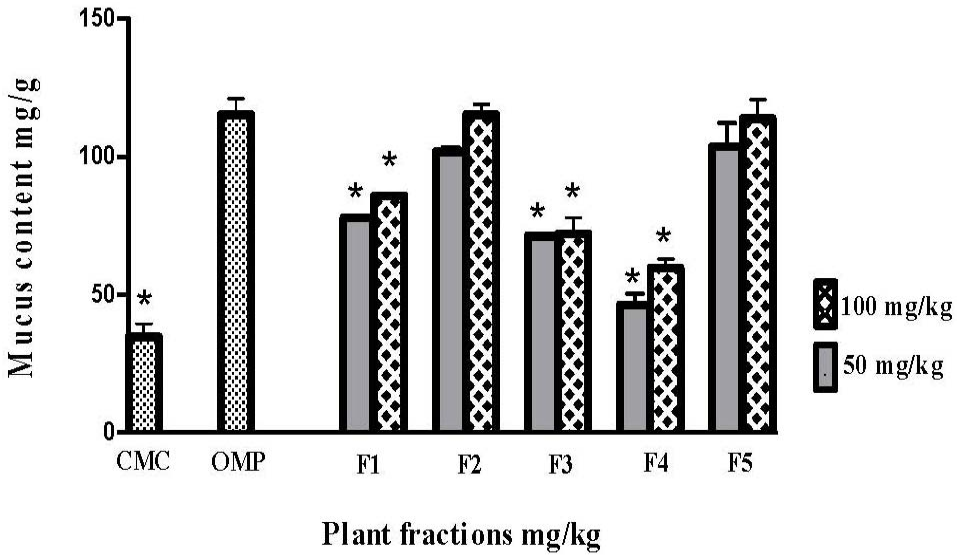
Effect of the fractions obtained from *Polygonum minus* on the mucus content in gastric tissue. Data are expressed as mean ± SEM (*n* = 6) * significantly different in comparison to omeprazole, *p* < 0.05 using one-way ANOVA.

Gastric mucus wall production after pre-treatment with all the fractions was increased in a dose-dependent matter, as shown in Figure 4. The animals which were treated with 100 mg/kg F2 and F5 showed increased production of mucus (115.29 mg/g ± 3.69 and 113.8 mg/g ± 6.85, respectively); there was no significant difference (*p* < 0.05) between these groups and the omeprazole positive control group.

### 2.3. Effect of *Polygonum minus* Fractions on PGE2 Synthesis

**Figure 5:**
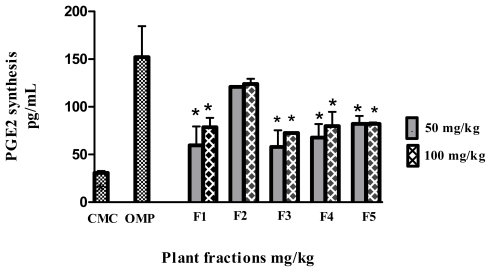
Effect of the fractions obtained from *Polygonum minus* on the PGE2 synthesis level in gastric tissue. Data are expressed as mean ± SEM (*n* = 6) * significantly different in comparison to omeprazole, *p* < 0.05 using one-way ANOVA.

The PGE2 synthesis level increased significantly (*p* < 0.05) in all groups compared to the CMC negative control group. The F2 pre-treated group exhibited no significant difference with the PGE2 synthesis (123.9 pg/mL ± 5.52) in comparison with the omeprazole positive control group (152.2 pg/mL ± 32.45; Figure 5).

### 2.4. Effect of the *Polygonum minus* Fractions on the Hexosamine Level

**Figure 6:**
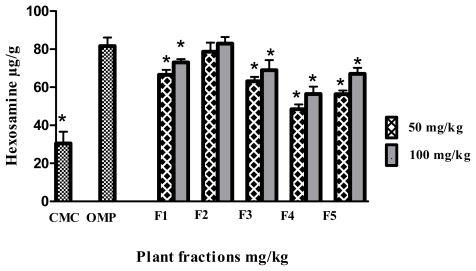
Effect of the fractions obtained from *Polygonum minus* on the hexosamine content in gastric tissue. Data expressed as mean ± SEM (*n* = 6). * significantly different in comparison to omeprazole, *p* < 0.05 using one-way ANOVA.

All the fractions markedly showed high levels of hexosamin, and they are significantly (*p* < 0.05) different compare to CMC negative control group. The rats pretreated with F2 (82.95 μg/g ± 3.45) showed highest level of hexosamin and there is no significant difference (*p* < 0.05) with omeprazol positive control group (81.60 μg/g ± 5.41) (Figure 6).

The ulcer prevention activity of phenolic compounds and their derivatives, notably demonstrated in various gastric induction models, have been postulated to occur by mechanisms that include the stimulation of prostaglandin synthesis, the elevation of mucus production and the reduction of gastric pH [28]. Accordingly, the PGE2 synthesis level was determined in gastric tissue homogenates; sustained PGE2 levels were observed in the case of treatment with omeprazole, a reference drug used as an antiulcer intervention. Furthermore, the mucus barrier is an important agent in ulcer protection [29]. In the present research, (Figure 4) shows that F2 promoted better mucus production compared to the other fractions. PGE2 is a main stimulant of mucus production in the stomach, and this finding is in agreement with a previous report [30]. Besides PGE2 and its relation to mucus production, hexosamine, which is the main glycoprotein of mucous tissue in the stomach, was positively correlated with mucus production. Figure 6 demonstrates that F2, which was associated with high mucus production, promoted high levels of hexosamine. These results agree with a previous study reported by Bhattacharya, *et al*.[31].

### 2.5. Effect of *Polygonum minus* Fractions on Superoxide Dismutase (SOD) Activity

**Figure 7:**
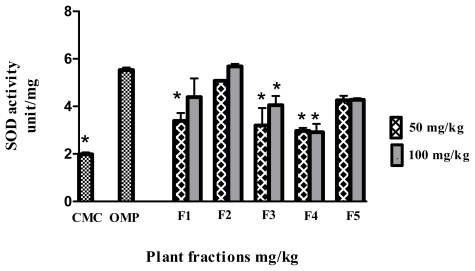
Effect of the fractions obtained from *Polygonum minus* on the activity of superoxide dismutase (SOD) in the gastric mucosa. Data are expressed as mean ± SEM (*n* = 6). * significantly different in comparison to omeprazole, *p* < 0.05 using one-way ANOVA.

Enzyme scavenging activity, particularly via SOD, as presented in Figure 7, showed that F2 (5.68 ± 0.1 unit/mg) and F5 (4.27 ± 0.06 unit/mg) significantly (*p* < 0.05) promoted scavenging activity against ethanol-induced ulcers. In contrast, F1 at 50 mg/kg, as well as F3 and F4 at both treated doses showed significantly different results (*p* < 0.05) compared to the omeprazole treated group.

In addition, we assessed the antioxidant enzyme level as a gastroprotection mechanism. As mentioned previously, oxidative stress is one of the main causes of gastric ulcers induced by ethanol. Hence, antioxidants are hypothesized to be one of the prevention factors against ethanol-induced ulcers. Previous work showed the powerful *in vitro* antioxidant activity of *P. minus* [7,32]. Therefore, this study examined the activity of an *in vivo* enzymatic scavenger such as SOD. This enzyme is considered as the first line of defence against reactive oxygen species [22]. The results show that pre-treatment with F2 promoted ulcer prevention and was associated with higher levels of SOD compared to omeprazole. On the other hand, pre-treatment with F3 and F4, with a low ulcer inhibition percentage, showed a reduction in the SOD level which may have been partly responsible for oxidative damage to the stomach tissue.

### 2.6. Phytochemical Analysis

**Figure 8:**
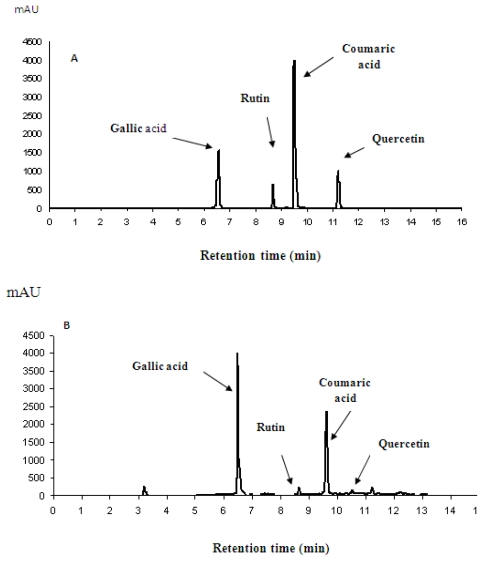
(**A**) HPLC chromatogram of the standards: gallic acid (6.43 min), rutin (8.60 min), coumaric acid (9.43 min) and quercetin (11.13 min) and (**B**) F2 with the same retention times as the standard chemicals.

The phytochemical analysis were performed using HPLC and UPLC-ESI-MS/MS, High performance liquid chromatography analysis determined the phenolic compounds contained in the ethanolic extract of *P. minus* fraction (F2). It was shown that F2 was the fraction with highest amount of phenolic compounds such as gallic acid, rutin, coumaric acid and quercetin as presented in Figure 8B. After comparing the peaks to those of the standard (Figure 8A), gallic acid was found to be the main active compound, followed by coumaric acid. The results were confirmed by UPLC-ESI-MS/MS, and these data were matched with the theoretical results available in our lab.

## 3. Experimental

### 3.1. Plant Extraction and Fractionation

Dried leaves of *P. minus* were obtained from Ethno Resources Sdn Bhd, Selangor Malaysia, The plant was identified, and voucher specimen was kept in our laboratory for future references. Air-dried powdered leaves of *P. minus* were extracted by drenching in 95% ethanol (50 g/1000 mL, w/v) at room temperature for 3 days. Afterwards, the solvent was filtered, followed by concentration under reduced pressure until excess solvent was removed. The resulting yield was 5.8 g/100 g. Next, 1.5 g of the crude extract was chromatographed over silica gel (70–230 mesh, ASTM Merck KGaA) using FLEX™ columns. Different fractions were obtained from the stepwise elution of different ratios of hexane, ethyl acetate, methanol, acetonitrile, and distilled water. Depending on the TLC profile, similar fractions were combined. Ultimately, five fractions were obtained from *P. minus* leaf extract. The first fraction, F1, was eluted with hexane:ethyl acetate 1:1 v/v to give 0.267 g. The second fraction, F2, was obtained using ethyl acetate:methanol 1:1 v/v and gave 0.651 g. The third fraction, F3, was eluted with methanol:acetonitrile 1:1 v/v and gave 0.241 g. The fourth fraction, F4, was eluted with acetonitrile:distilled water 1:1 v/v yielding 0.20 g1:1 v/v. Finally, the fifth fraction, F5, was eluted with distilled water producing 0.12 g1:1 v/v. The gastroprotective ability of all of the fractions was evaluated in order to identify the most active fraction.

### 3.2. Experimental Protocol for Anti-Ulcer Evaluation

Following the method described by Garg, *et al*. [33] with minor modifications, healthy male Sprague Dawley rats (150–200 g) were deprived of food for 48 h before the experiment was conducted, but were allowed free access to drinking water up until 2 h before the experiment. All rats were treated by orogastric intubations. The animals were divided randomly into seven groups of six rats per each. The first group, the negative control group, received carboxymethyl cellulose CMC, 0.25% w/v, 5 mL/kg. The second group, which was the positive control group, received 20 mg/kg of omeprazole 5 mL/kg. The remaining groups, the treated groups, were pre-treated with 50 and 100 mg/kg of the fractions obtained from the ethanolic extract of *P. minus*: F1, F2, F3, F4, and F5, respectively. After one hour, all rats were administered 95% ethanol 5 mL/kg and after an additional 60 min, the rats were sacrificed by an overdose of ketamine 100 mg/mL. The stomach was rapidly removed for gross and histological evaluation and biochemical testing.

### 3.3. Evaluation of Gross and Histological Changes

Macroscopically, the lesions appeared as hemorrhage bands along the long axes of the stomach. The lesion of each stomach was calculated under a dissecting microscope (1.8×) using a hemocytometer (big square: length × width = 10 mm × 10 mm = ulcer area). The sum of the areas of all lesions for each stomach was implemented in the calculation of the ulcer area (UA) wherein the sum of small squares × 4 × 1.8 = UA (mm^2^) as described by Njar and Mahmood, *et al.* [34,35]. The ulcer inhibition percentage (I%) was calculated according to the following formula:

[(I%)=[(UA control-UA treated)÷UA control]×100%]

Following the assessment of the ulcer score, stomachs were fixed in a buffered formalin (10%) solution for histological examination. The fixed stomachs were processed, sectioned every 5 μm and stained with H & E.

### 3.4. Determination of Gastric Wall Mucus

Gastric wall mucus was determined according to the procedure of Corne and Al-Qarawi, *et al.* [36,37]. The glandular portion of the stomach was removed, weighed and incubated in tubes containing 10 mL of 0.1% Alcian blue solution for 2 h, after which the excess dye was removed by two successive rinses with 10 mL of 0.25 M sucrose. Each stomach was extracted with 10 mL of 0.5 M magnesium chloride by intermittent shaking for 1 min at 30 min intervals for 2 h. The resultant solutions were vigorously shaken with an equal volume of diethyl ether and the emulsions were then centrifuged at 3000 g for 10 min. The absorbency of the aqueous layer was measured against a buffer blank at 580 nm. The quantity of blue dye recovered per gram of wet glandular tissue was then calculated from a standard curve.

### 3.5. Preparation of Stomach Tissue Homogenate

The stomachs were homogenized using ice cold Tris-HCl pH 8.2 (1 g in 10 mL) on ice. The homogenate tissues were centrifuged at 4500 g for 15 min at 4 °C, and then stored at −80 °C until they were used. In this study, the homogenate was analyzed in order to estimate superoxide dismutase, hexosamine and prostaglandin synthesis.

#### 3.5.1. Determination of Prostaglandin Synthesis

Prostaglandin E2 (PGE2) levels produced in the gastric mucosa were measured following the procedure described in Cayman’s Protein Determination Kit.

#### 3.5.2. Determination of Total Hexosamine

The concentration of hexosamine in stomach tissue was analysed according to the reported method of [38] with minor modifications. The hydrolyzation of stomach tissue was carried out using 6 M HCl, then neutralized using 6 M NaOH. Next, 0.5 mL of freshly prepared acetyl acetone was added to 0.5 mL of the neutralized samples, and the mixture was boiled at 100 °C for 15 min. Following cooling of the mixture, 0.5 mL of Ehrlich reagent was added and the absorbance of colored chromogens was calculated at 530 nm using a UV 1601 spectrophotometer (Shimadzu-Japan).

#### 3.5.3. Determination of Superoxide Dismutase (SOD)

Stomach superoxide dismutase activity was measured at 460 nm using a plate reader following the method reported in Cayman’s Superoxide Dismutase Assay Kit.

### 3.6. Phytochemical Analysis

#### 3.6.1. HPLC

Phytochemical analysis was conducted to identify phenolic compounds by using the high performance liquid chromatography (HPLC) method, using an LC-20AD apparatus (Shimadzu Corp, Kyoto, Japan) [39]. The experiments were carried out using a reversed phase C18 Hypersil GOLD column (250 mm × 4.6 mm; Thermo Fisher Scientific). The samples were eluted with a stepwise elution of the mobile phase as described in [40] with slight modifications. The mobile phase consisted of solvent A (H_2_O + 0.1% TFA) and solvent B (methanol + 0.1% TFA), at a unique flow rate of 1 mL/min. The gradient elution program was as follows: 0 min 90% A:10% B; 5 min 55% A:45% B; 20 min 50% A:50% B; 21 min 40% A:60% B; 22 min 90% A:10% B. The sample injection volume was 20 μL followed by equilibration run of 15 min and monitored with an SPD-20 A (UV) at 280 nm.

#### 3.6.2. UPLC-ESI-MS/MS

The phenolic profiles were further confirmed by ultra-performance liquid chromatography-electrospray tandem mass spectrometry. This method is described in detail by Chua, *et al.* [41]. To perform analytical UPLC, a Waters Acquity (Milford, MA, USA) system was coupled to a triple quadrupole-linear ion trap tandem mass spectrometer (Applied Biosystems 4000 Q TRAP; Life Technologies Corporation, Carlsbad, CA, USA) with an electrospray ionization (ESI) source.

### 3.7. Statistical Analyses

The data were analyzed using Statistical Package Social Science (SPSS), version 17.0 [42]. One-way ANOVA was used to show the mean differences between all samples (* *p* < 0.05) using GraphPad PRISM^®^, version 5.0 [43].

## 4. Conclusions

**Scheme 1:**
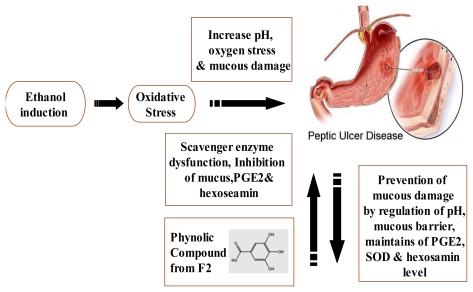
The phenolic compounds in F2 prevent peptic ulcer generation by reactive oxygen species, particularly ethanol, by regulating mucous production, and maintaining PGE2, SOD and hexosamine levels.

The ethyl acetate:methanol fraction 1:1 (F2) is the best fraction for gastroprotective activity against oxidative stress caused by ethanol induction model. The efficacy of F2 is based on its high content of phenolic compounds, particularly gallic acid and coumaric acid, which contribute to antioxidant activity, mucus barrier as well as maintenance of PGE2 and SOD levels (Scheme 1). This confirms the traditional use of *P. minus* for its gastroprotective capability and it can therefore be formulated and recommended to be a therapeutic drug for gastric ulcer.
